# Frontiers in Global Mangrove Forest Monitoring

**DOI:** 10.3390/rs15153852

**Published:** 2023-08-03

**Authors:** Chandra Giri

**Affiliations:** Office of Research and Development, United States Environmental Protection Agency, 109 T.W. Alexander Dr., Durham, NC 27713, USA

Enhanced spatial, spectral, thematic, and temporal resolution is imperative to optimize the monitoring of mangrove forests, ensuring their effective conservation and management as crucial global resources [1]. These forests play a vital role in providing invaluable ecosystem goods and services to both human society and nature. A prime example of their significance lies in their exceptional ability to sequester carbon, surpassing that of other tropical forests, thus making a substantial contribution towards mitigating the climate change crisis. Furthermore, these forests act as natural protective barriers, safeguarding coastal communities from the destructive forces of hurricanes and tsunamis.

Unfortunately, mangrove forests are under threat due to both natural and anthropogenic forces. Currently, the conversion of mangroves to alternative land uses stands as the dominant factor driving these changes. However, the increasing prominence of sea level rise, global warming, and the intensification of natural disasters, such as hurricanes, is likely to play a more significant role in the future. Thus, observation and monitoring of mangrove distribution and their dynamics are crucial for understanding and addressing the impacts of these changes along with their broader ecological implications for both society and nature. Such information is also needed to manage these forests on a sustainable basis.

The utilization of remote sensing technology is of utmost importance in accurately mapping and monitoring mangrove forests across various scales, ranging from local to global scales. The advancement of this invaluable tool enables us to achieve an enhanced spatial, spectral, thematic, and temporal resolution, thereby significantly improving our scientific comprehension of these expansive ecosystems. Over the past two decades, the availability of new and higher resolution satellite data has played a pivotal role in advancing our knowledge in this field. Moreover, there have been notable advancements in methodologies, computing technologies, and data interpretation expertise, further enhancing our capabilities. As a result, the acceptance and recognition of remote sensing-derived findings by a broader community have also witnessed a substantial improvement.

*Remote Sensing* is pleased to publish the second volume of a Special Issue dedicated to the observation and monitoring of mangroves using remote sensing techniques. This Special Issue covers a wide range of applications, including the utilization of optical and radar data. Researchers have employed state-of-the-art techniques for data acquisition, management, exploitation, processing, and analysis of remote sensing data in the context of mangrove forest applications. Contributions in the form of eleven research papers and one review paper have been included in this volume.

In the first paper of this Special Issue, Purwanto et al. utilized decision trees and random forest classification algorithms to map and monitor one of the most extensive mangrove forests in Indonesia. The mangrove forests in the Sembilang National Park were facing threats from human activities, necessitating rapid mapping and monitoring as a result. These authors conducted a change analysis from 2002 to 2019 using the decision tree and random forest algorithms. They employed secondary data sources, such as the multi-error-removed improved-terrain digital elevation model (MERIT DEM) accompanied with existing mangrove maps, along with various indices, like the normalized difference moisture index (NDMI) and the normalized difference soil index (NDSI). This study compared the performance of the classification algorithms for the interpretation of the Landsat 7 ETM+ and Landsat-8 OLI data. Their results indicated that the decision tree algorithm with the parameter combination of NDMI, NDSI, and DEM effective for the classification of Landsat-7 ETM+. The random forest classification algorithm outperformed the decision tree algorithm in mapping mangrove forests, as all the parameters of the random forest model exhibited a higher producer accuracy.

Vul et al. conducted a change analysis in three provinces on the northern coast of Vietnam, namely Thai Binh, Nam Dinh, and Hai Phong, using multi-temporal Landsat imagery. This study aimed to monitor the dynamic nature of mangrove forests in this region, focusing on the time period between 1990 and 2022. These researchers employed the Google Earth Engine (GEE) cloud computing platform. The overall accuracy obtained in the year 2022 was 91.98%, while the Kappa coefficient was 0.84. Their results also revealed fluctuations in the mangrove area over time, with periods of decline and elevation. However, since 2005, mangrove forests have exhibited a continuous increase, mainly due to the implementation of restoration programs and policies by the Vietnamese government and local authorities. Notably, this study demonstrated the potential of Landsat time series imagery, pixel-based algorithms, and the GEE platform for the long-term monitoring of mangrove forests.

Ghosh et al. conducted a multiscale diagnosis of mangrove status in a data-poor context using very high spatial resolution (VHSR) satellite images in the Pichavaram mangrove forest, located in Tamil Nadu, India. Pichavaram mangrove forests face threats from cyclones as well as reduced freshwater flow from upstream sources. This study analyzed the changes in the mangrove area spanning from the years 2003 to 2019 at a spatial resolution of 4 m. Specifically, they employed Quickbird (QBD) images in the years of 2003 and 2005 (that were captured shortly after the Indian Ocean tsunami which significantly impacted the region on 26 December 2004), along with GeoEye-1 (GEO) images that were obtained in 2011 and 2016, and a Worldview-3 (WV3) image from 2019. They classified the mangrove and non-mangrove areas using supervised classification available in ERDAS Imagine processing software. Accuracy assessment was performed for each year, achieving an overall accuracy of 85% or higher. Post-classification change analysis was conducted for both natural and planted mangroves between 2003 and 2019, with their results revealing that the mangrove forest area experienced a 28.0% increase (201.2 hectares) from 2003 to 2019. The expansion of these mangrove areas predominantly resulted from the conversion of non-mangrove areas into mangrove habitats. These researchers concluded that despite the limited availability of ground-truth data, VHSR data proved to be valuable in providing a multiscale diagnosis of this ecosystem’s condition.

Bunting et al. emphasized the work performed by the Global Mangrove Watch (GMW) in updating the extent of mangrove forests from the year 2010. These researchers highlighted that the latest version of the GMW, namely GMW v2.5, represents a significant improvement over the previous version. They identified 204 regions that were either inaccurately mapped or completely missing from the previous maps, and these areas were updated accordingly. The primary objective of this endeavor was to enhance the existing map through the incorporation of new information. To achieve this, they employed the XGBoost binary classification algorithm for their classification process. As a result of this revision, an additional 2660 square kilometers of mangroves were added, leading to a revised global mangrove extent equivalating to approximately 140,260 square kilometers for the year 2010. However, it is important to note that this study did not examine for any potential forest loss in other areas, which thereby presents a significant limitation of this research.

Rahmandhana et al. conducted mangrove species mapping based on spectral reflectance using extremely high-resolution satellite data from WorldView 2. This study was carried out in the Karimunjawa and Kemujan Islands, located in the central Java province of Indonesia. To create a detailed inventory of mangrove forest biodiversity, particularly in Java, where the diversity of mangrove species is exceptionally high, the utilization of very high-resolution imagery for species mapping in this context was not only possible, but also essential. The study area was found to house approximately 44 species, including 25 true mangroves and 19 mangrove associates. To perform species mapping, these researchers employed the spectral angle mapper (SAM), spectral information divergence (SID), and spectral feature fitting (SFF) algorithms. Field data, such as mangrove species identification, coordinate locations of targeted mangrove species, and spectral reflectance measurements of mangrove species using a field spectrometer, were all collected. Dendrogram analysis utilizing the Ward linkage method was conducted to classify mangrove species based on their distances between clusters of spectral reflectance patterns. This classification process resulted in two, four, and five species groups for Levels 1 to 3, respectively, while individual species were identified under Level 4. The SID algorithm achieved the highest overall accuracy, while the SFF algorithm yielded the most accurate results for mapping individual species. As they expected, these findings indicated that as the number of classes to be mapped increased, the mapping accuracy consequently decreased.

Zhu et al. conducted a spatiotemporal simulation of mangrove forests under different scenarios in Hainan Island, China. Their study utilized a total of 12 drivers, including the elevation, slope, enhanced vegetation index (EVI), EVI change trends, distance to major roads, distance to minor roads, distance to the sea, distance to rivers, distance to aquaculture ponds, distance to building land, distance to suitable land for mangroves, and a spatial autocorrelation factor, for the simulation. Various models, such as logistic regression, support vector regression, and the random forest model, were compared with one another in terms of their ability to capture the spatial characteristics of mangrove forests. Three development scenarios were established: a natural growth scenario (NGS), an economic development scenario (EDS), and a mangrove protection scenario (MPS). The CLUE-S model was employed to predict the spatiotemporal distribution of mangrove forests from the years 2022 to 2037 under these different scenarios. Subsequently, based on the prediction results, the future change trends of mangrove forests from 2017 to 2037 were analyzed. The simulation results of these different models demonstrated that AutoRF (random forest with spatial autocorrelation) performed the best in simulating the spatial characteristics. Importantly, specific factors, such as the enhanced vegetation index (EVI), various location indices, and the spatial autocorrelation factor were all found to significantly improve the accuracy of the mangrove simulations. The prediction results for Hainan Island indicated that under the NGS, the mangrove area would experience slow growth, while under the EDS, it would decrease significantly. Conversely, the mangrove area would increase significantly under the MPS. Based on these findings, the MPS was identified as the most suitable development direction for the future, as it offers a balanced approach that promotes economic development while ensuring mangrove preservation.

Chambarlain et al. utilized Landsat dense time series data to monitor the mangrove forest cover and its phenology in Central Queensland, Australia. Although the exploitation of mangroves by coastal communities is strictly regulated in Australia, these forests are still being threatened with landscape modifications and hydrological alterations upstream. This study employed Landsat reflectance data from the years 2009 to 2019 to track mangrove forest cover changes. To address the persistent cloud cover issues impacting certain areas, mosaics of three-year windows were used to generate cloud-free mosaics. Analysis was conducted using the Google Earth Engine and a random forest classifier. Additionally, secondary information, such as the shuttle radar topography mission (SRTM) and previously established mangrove land cover maps, were incorporated. The overall classification accuracies and Kappa coefficient for the land cover maps in the years 2008–2010 and 2018–2020 were both determined to be 95%. Furthermore, a decrease of 1480 hectares (−2.31%) in mangrove coverage was also observed from 2009 to 2019. Extending from this, an examination of intra- and inter-annual seasonality in mangrove growth phenology was conducted using an NDVI-based time series. Linear and harmonic regression models, as well as TIMESAT metrics, were employed to analyze mangrove forests in three sections of the study region. Together, these findings indicated a correlation between the growth phenology of mangrove forests, precipitation anomalies, and the occurrence of severe tropical cyclones over the time series.

Niu et al. conducted a global sensitivity analysis for the canopy reflectance and vegetation indices of mangroves. This study focused on a one-dimensional canopy reflectance model to systematically analyze the sensitivity of mangroves to various biophysical and environmental factors. Different scenarios were created, including sparse and dense canopies, to assess the impact of these factors on simulated canopy reflectance spectra and selected Sentinel-2A vegetation indices. This study employed a variance-based method and a density-based method to compare the computed sensitivity indices. The results revealed that the fractional cover and leaf-to-total area ratio of mangrove crowns were among the most influential factors across all examined scenarios. These findings highlight the significant role of these factors in shaping the canopy reflectance and vegetation indices of mangroves.

Ghorbanian et al. utilized Sentinel-1 and Sentinel-2 satellite data to map the mangrove ecosystem in the Hara protected area in Qeshm, Iran. The analysis was conducted on the Google Earth Engine at a spatial resolution of 10 m. The data acquired using these Sentinel satellites in 2019 were utilized to generate a composite of optical and synthetic aperture radar (SAR) data. To classify the different components, a pixel-based random forest (RF) classifier was employed. This study successfully generated six distinct classes: mangrove, mudflat, deep water, tidal zone, shallow water, and aerial roots. The resulting mangrove ecosystem map exhibited a high accuracy, with an average overall accuracy of 93.23% and a Kappa coefficient of 0.92. These findings demonstrate the reliability and effectiveness for mapping mangrove ecosystems using Sentinel-1 and Sentinel-2 satellite data.

Kanniah et al. utilized the MODIS-derived leaf area index (LAI) and gross primary productivity (GPP) to investigate the fragmentation of mangrove forests in the southern region of Peninsular Malaysia, specifically in Iskandar Malaysia. This study aimed to assess the impact of land cover changes, including urbanization, plantations, and aquaculture activities, on the mangrove forest ecosystem. The results revealed a decline in the mangrove forest area due to land cover changes. However, the areas that remained undisturbed revealed an increase in both the mean LAI and GPP, signifying the mangrove forest’s capacity to absorb CO_2_ when left undisturbed. Furthermore, areas that experienced mangrove loss but were replaced with oil palm plantations exhibited a decrease in the mean LAI. The fragmented mangrove patches also showed an increase in GPP, potentially due to the smaller patch sizes (<9 ha) and the edge effects, which promote higher levels of productivity in areas exposed to abundant solar radiation along the patch edges. The impact of fragmentation on the GPP was found to depend on the type of land transformation along with patch characteristics, such as size, edge, and shape complexity.

Muang and Sasaki conducted a study to evaluate the natural recovery of mangroves in the Wunbaik mangrove forests of Myanmar following human disturbance, specifically focusing on the abandoned shrimp ponds. These researchers utilized cloud-free Sentinel-2 images that were captured on 21 January 2020 and 23 December 2015, both during the dry seasons. They employed an artificial neural network (ANN) classification approach with a transfer learning method to classify these two dates’ images. Additionally, a post-classification change analysis approach was employed to assess the changes that occurred. To identify the naturally recovering mangroves, three abandoned shrimp ponds were selected based on field investigations and their change detection results were then extracted. The proposed methodology achieved a high level of accuracy, with an overall accuracy of 95.98% and a Kappa coefficient of 0.92 for the 2020 classification. For the 2015 prediction, the transfer learning approach improved the model’s performance, resulting in an overall accuracy of 97.20% along with a Kappa coefficient of 0.94. The analysis of the change detection results revealed a slight decrease of mangrove forests within the Wunbaik mangrove forests between the years 2015 and 2020. However, naturally recovering mangroves were identified in approximately 50% of each abandoned site within a relatively short abandonment period. These findings contribute to our understanding of the natural regeneration process of mangroves following human disturbance, specifically in abandoned shrimp ponds.

In the final paper of this Special Issue, Tran et al. presented a comprehensive review paper on spectral indices for mangrove remote sensing. This study covered the period between 1996 and 2021, examining the range of spectral indices that have been developed and utilized in the context of mangrove remote sensing. This review revealed that spectral indices have been predominantly used for various aspects of mangrove analysis, excluding the identification of mangrove species. The aspects that were covered included the mangrove extent, their distribution, and above-ground parameters (such as the carbon density, biomass, canopy height, and leaf area index estimation), as well as changes in these aspects over time. Among the spectral indices, the NDVI emerged as the most widely applied, appearing in 82% of the reviewed studies, followed by the EVI, which was noted as being used in 28% of the studies examined. While the development and application of potential indices for characterizing mangrove cover have shown growth (with six indices having been published so far), the NDVI remains the most popular index for mangrove remote sensing. However, this review also highlights the limitations and gaps that are present within current studies. Considering the digital era, the authors suggested future directions for research, emphasizing the need to explore spectral index applications in connection with time series imagery and the fusion of optical sensors for more comprehensive and accurate mangrove studies.

In summary, the utilization of cloud computing platforms for data analysis in mangrove research is growing. Researchers benefit from the flexibility, cost savings, enhanced security, scalability, and improved collaboration offered by cloud computing. There is a rising demand for mapping mangroves at higher resolutions, specifically at levels higher than 5 m. Compared to the past, supervised classification approaches are now more commonly used instead of unsupervised classification. The most popular application used in the remote sensing of mangroves is the mapping of mangrove and non-mangrove areas and conducting time series change analysis. Both optical and synthetic aperture radar data are currently being employed for mangrove mapping. Overall, researchers are generating higher resolution mangrove data and producing improved results for change analysis, ranging from local to global scales. This progress has been made possible with the increased availability of free and higher resolution remote sensing data, advancements in computing technology (both hardware and software), growing collaborative efforts among researchers, increased availability of expertise, better understandings of mangrove forests, and the development of improved methodologies.
